# A Novel Cytomegalovirus-Induced Regulatory-Type T-Cell Subset Increases in Size During Older Life and Links Virus-Specific Immunity to Vascular Pathology

**DOI:** 10.1093/infdis/jit576

**Published:** 2013-11-07

**Authors:** Nadia Terrazzini, Martha Bajwa, Serena Vita, Elizabeth Cheek, David Thomas, Nabila Seddiki, Helen Smith, Florian Kern

**Affiliations:** 1 Division of Medicine; 2 Division of Primary Care and Public Health, Brighton and Sussex Medical School; 3 School of Computing, Mathematical and Information Science, University of Brighton, Brighton; 4 School of Health, Sport and Bioscience, University of East London, London, United Kingdom; 5 Department of Public Health and Infectious Diseases, Institute Pasteur, Cenci-Bolognetti Foundation, University Sapienza of Rome, Italy; 6 INSERM U955 Equipe 16, Paris; 7 Université Paris-EstCréteil; 8 Vaccine Research Institute, Créteil, France

**Keywords:** Arteriosclerosis, infection, immunology, inflammation, hypertension, iTreg, Regulatory T-Cells

## Abstract

***Background.*** Cytomegalovirus (CMV) infection directly targets vascular endothelium and smooth muscle and at older ages is associated with accelerated vascular pathology and mortality. CMV-specific cellular immunity might directly contribute to this process.

***Methods.*** Conventional ex vivo activation–induced T-cell responses to 19 dominant CMV antigens, along with CMV-specific inducible regulatory-type CD4^+^ T cells (iTregs), were measured in healthy older people, using a novel protocol that included classic Treg markers alongside the activation marker CD134. Measurements were correlated with diastolic, systolic, and mean arterial blood pressure, a surrogate marker for arterial stiffness.

***Results.*** CMV-specific iTregs recognized the same antigens as conventional CD4^+^ T cells and were significantly more frequent at older ages. They suppressed antigen-specific and nonspecific proliferation and in large part expressed Foxp3. Frequencies of CMV-specific iTregs and CD8^+^ T cells (summated response) were significantly associated with diastolic and mean arterial pressures. Confounders, including age, body mass index, smoking, antihypertensive medication use, or C-reactive protein levels, did not explain these observations.

***Conclusions.*** A novel CMV-induced regulatory-type CD4^+^ T-cell subset is readily detectable in CMV-infected people and, like the aggregate CD8^+^ T-cell response to the most dominant CMV antigens, is quantitatively associated with arterial stiffness in older life. Whereas CD8^+^ effector T cells might directly cause vascular injury, iTregs may attenuate this response.

Since the 1990s, cytomegalovirus (CMV) infection has been implicated in vascular pathology and immunosenescence [1–5]. CMV directly targets endothelial and smooth muscle cells and is found in vascular lesions, where it might, directly or indirectly, contribute to pathology [3–13]. Moreover, after onset of CMV infection, endothelial cells upregulate class I major histocompatibility complex (MHC) molecules, rendering them more vulnerable to CMV-directed cytotoxicity [14, 15].

In the context of human immunodeficiency virus (HIV) infection, immunosenescence and vascular pathology are observed at earlier ages [16], and associations of these conditions with CMV/HIV coinfection have been subject to intense investigation recently [17]. A number of clinical studies have suggested a role of CMV-specific T-cell immunity in vascular changes. For example, Parrinello et al [18] reported that higher anti-CMV antibody titers are associated with carotid artery lesions in HIV-positive individuals who became aviremic after they initiated highly active antiretroviral therapy, implying that T-cell reconstitution contributes to this association. Adding more concrete evidence of T-cell involvement, Hsue et al [19] observed that that carotid intima media thickness is directly related to the size of a CMV protein–specific CD8^+^ T-cell response in both HIV-positive and HIV-negative individuals. It appears from the literature to date that a role of CMV-specific CD8^+^ effector T cells in causing or aggravating vascular injury is highly likely. However, the role of CD4^+^ T cells is still very unclear.

Among CD4^+^ T cells, inducible regulatory T cells (iTregs) are of specific interest, because they are thought to be induced by the same antigens as conventional T-cell responses and may be involved in attenuating the immune response. In hepatitis, for example, iTregs are thought to protect vital tissue but also reduce viral clearance and so promote chronic infection [20, 21].

After onset of CMV infection, iTregs might limit acute vascular damage caused by effector T cells. This would be in agreement with finding in a recent publication demonstrating that Tregs can attenuate atherosclerosis in a mouse model [22]. Unfortunately, no specific marker of iTregs is available for tracking them in the same way as Tregs more generally.

Here, we aimed to identify iTregs by combining the classic Treg markers CD25 and CD39 [23, 24] with a short-term activation marker, CD134 (OX40) [25]. Whereas the expression of CD25 and CD39 converges on strongly suppressive Tregs, coexpression of CD25 and CD134 following 48 hours of antigen stimulation identifies antigen-specific, activated CD4^+^ T cells [26]. The goal of combining all 3 markers was to identify iTreg-type CD4^+^ T cells and, to be comprehensive, we used a range of different CMV antigens for stimulation (CMV lysate and 6 different peptide pools spanning the CD4 target proteins UL83, UL55, UL86, UL99, UL153, and UL32) [27, 28]. To capture CMV-specific CD4^+^ and CD8^+^ effector T-cell responses at the same time, we additionally studied the short-term activation markers CD154 upregulation, degranulation, and interleukin 2 (IL-2), tumor necrosis factor α (TNF-α), and interferon γ (IFN-γ) production among CD4^+^ and CD8^+^ T cells following overnight stimulation in a uniquely comprehensive approach, using the aforementioned 6 plus 13 additional CMV target antigens (Table 1) [28].

**Table 1.** Custom Peptide Pools Used for T-Cell Stimulation

Pool	Protein(s)^a^	Peptides, No.
1	UL55^b^	224
2	UL83^b^	138
3	UL86^b^	340
4	UL122	120
5	UL123	143
6	UL99^b^	45
7	UL153^b^	67
8	UL32^b^	260
9	UL28	92
10	UL48A	281
11	UL48B	281
12	US3	44
13	UL151; UL82	82; 137
14	UL94; US29	84; 113
15	UL103; US32	60; 43
16	US24; UL36	123; 117

^a^ Responses to pools 1 through 16 (covering 19 cytomegalovirus proteins) were added to derive summated CD4^+^ and CD8^+^ T-cell responses.

^b^ Dominant CD4^+^ T-cell target protein [28].

CMV-specific CD4^+^ T cells matching our definition of iTregs were common in CMV-infected people and displayed the hallmark features (phenotype and function) of Tregs. Their levels were significantly increased in older people, particularly older women, and correlated with levels of CD8^+^ effector T cells. Linear regression analysis including pertinent covariates such as age, body mass index (BMI; defined as the weight in kilograms divided by the height in meters squared), blood pressure medication use, smoking, and inflammation (ie, C-reactive protein [CRP] levels) demonstrated a significant association between CMV-specific T-cell subsets (CD8^+^ T cells and iTregs) and diastolic blood pressure, as well as mean arterial blood pressure, which is an established surrogate marker for vascular stiffness [28].

## METHODS

### Donors

We recruited 131 healthy older volunteers (age, 60–85 years) from general practices and 55 healthy young people (age, 20–35 years) from a population of university students and staff (>90% of study participants were white, and the remainder were black African or Asian). After a participant provided written informed consent, a trained research nurse recorded vital signs, including heart rate and body temperature. A standardized brachial blood pressure measurement was performed for each participant after they had rested for 10–15 minutes in a seated position, using an Omron 705CP automated blood pressure monitor (Omron Healthcare, Milton Keynes, United Kingdom), after which 30 mL of blood was obtained from a cubital vein (additional details, as well as inclusion and exclusion criteria, can be found in the Supplementary Materials). The study was approved by the United Kingdom UK National Research Ethics Service.

### Routine Laboratory

CMV immunoglobulin G (IgG; Architect CMV IgG, Abbot, Maidenhead, United Kingdom) was measured in the Brighton and Sussex University Hospital (BSUH) virology laboratory; CRP (Cobas Tina-quant, CRP HS, Gen. 3, Roche Diagnostics, Mannheim, Germany) was obtained from the BSUH pathology laboratories.

### Reagents

Purified CMV lysate (Advanced Biotechnologies Inc, Columbia MD) was dissolved in dimethyl sulfoxide and used at a final concentration of 2 µg/mL. Phytohemagglutinin (PHA; Sigma-Aldrich, Gillingham, United Kingdom), the positive control for iTreg assays, and SEB (Source, Town, Country), the positive control for peripheral blood mononuclear cell (PBMC) assays, were used at final concentrations of 5 µg/mL and 1 µg/mL, respectively. Tuberculin (human tuberculin, SSI, Copenhagen, Denmark) was used at a final concentration of 10 µg/mL. Peptide pools (PepMix, JPT Peptide Technologies, Berlin, Germany) for each CMV protein (Table 1) were dissolved in dimethyl sulfoxide (Sigma-Aldrich) and used at a final concentration of 1 µg/mL per peptide for each pool.

### Whole-Blood and PBMC Activation Assays

For the present study, CMV-specific T-cell subsets were analyzed in 74 older and 39 younger randomly selected volunteers. Clinical details recorded for older volunteers are provided in Supplementary Tables 1 and 2. Whole blood was mixed at a ratio of 1:1 with complete medium, consisting of Roswell Park Memorial Institute (RPMI) 1640 medium supplemented with 100 U/mL penicillin, 100 U/mL streptomycin, 2 mM L-glutamine, and 10% fetal calf serum (Gibco, Life Technologies, Paisley, United Kingdom); stimulated with CMV lysate, CMV PepMixes, or PHA at the specified final concentrations; and incubated for 44 hours in a standard incubator.

PBMCs were isolated from heparin blood by density gradient centrifugation (Ficoll-Hypaque, PLUS Healthcare, Buckinghamshire, United Kingdom), washed twice with phosphate-buffered saline (PBS), and resuspended in complete medium. PBMCs (10^6^ cells per tube) were stimulated in the presence of anti-CD107 antibody, monensin (Golgistop, BD Biosciences), and one of the stimulants (positive control; one of 16 different PepMix solutions, CMV lysate, or tuberculin) for 2 hours before the addition of brefeldin A (Sigma-Aldrich). Incubation was stopped after an additional 14 hours.

Whole blood was stained with monoclonal antibodies to lineage markers CD3 and CD4 (BioLegend, Cambridge Bioscience, Cambridge, UK), Treg markers CD25 and CD39, and the activation marker CD134 (BD Biosciences) for 30 minutes at 4°C; fixed and lysed (fluorescence-activated cell sorter [FACS] lysing solution, BD Biosciences); and washed twice. PBMCs were surface stained with monoclonal antibodies to lineage markers (CD3, CD4, and CD8, BD Biosciences) and memory markers (CD27, BD Biosciences; and CD45RA, Beckman Coulter, High Wycombe, United Kingdom). They were then fixed and permeabilized using FACS lysing solution and FACS permeabilization solution 2, respectively (BD Biosciences); stained intracellularly for the activation markers IL-2, TNF-α, IFN-γ, and CD154; and washed twice. All samples were stored at 4°C in the dark before acquisition. Additional details are available in the Supplementary Materials.

### Cell Acquisition

Cells were analyzed on an LSRII flow cytometer, using FACSDiva software, version 6.1 (BD Biosciences), and FlowJo software, version 9 (TreeStar, Ashland, USA).

Separate gates were set for each activation marker on CD4^+^ and CD8^+^ T cells (ie, IL-2, degranulation, CD154, TNF-a, and IFN-γ). Boolean gates were used to identify functional combinations of markers. The sum of all Boolean gates represents the percentage of cells displaying at least 1 of the markers; these were termed “activated effector T cells” in this study. Whole-blood assays were analyzed with respect to CD25, CD39, and CD134 staining of CD4^+^ T cells, and Boolean gates were used for each marker as described above (Supplementary Figure 1). Values for unstimulated samples (negative control) were subtracted from those for stimulated samples for each individual gate.

### Suppression Assays

PBMCs were stimulated (for 44 hours at 37°C) with the UL83 peptide pool and stained with T-cell lineage and Treg markers. Antigen-induced iTregs (CD4^+^CD25^+^CD39^+^CD134^+^), nonresponding CD4^+^ T cells (CD4^+^CD25^−^), and CD3^−^ accessory cells were sorted (Aria-Icell-sorter, BD). After sorting, T-cell populations were rested in culture medium with IL-2 (100 IU/mL, Sigma-Aldrich), whereas accessory cells were stored at −80°C.

Responder cells were labeled with PKH67 (Sigma-Aldrich) according to the manufacturer's instructions, and 20 000 cells/well were plated in 96-well U-bottomed plates (Thermo Fisher Scientific, Nunc, Denmark). iTregs were added to responder cells at ratios of 1:1 and 1:10 (Tr:Te). A total of 50 000 accessory cells were added to each well. Cells were plated (200 µL/well) with 0.5 µg/mL soluble anti-CD3 OKT3 (BD Biosciences) or peptide-pool UL83 for restimulation. Positive (only responder cells, stimulant, and antigen-presenting cells) and negative proliferation controls (no restimulation) were run in parallel. On day 3, all cells were washed with FACS buffer (PBS, 0.5% bovine serum albumin, and 0.1% sodium azide) before staining. Proliferation of CD4^+^ responder cells was identified on the basis of decreasing PKH67 expression, as described elsewhere [29].

### Statistical Analysis

SPSS-20 was used for analysis. A 2-sided *P* value of < .05 was considered statistically significant. Non–log-transformed T-cell subset percentages were compared using the Mann-Whitney test for independent samples. Log transformation of T-cell subset percentages was used to improve the normality of the distributions. For the calculation of Pearson correlation coefficients (SPSS bivariate correlation procedure), log-transformed T-cell subset percentages were used (for correlations between T-cell subsets, as well as between T-cell subsets and diastolic blood pressure and mean arterial blood pressure). Unadjusted and adjusted linear regression models were used to evaluate factors associated with diastolic blood pressure and mean arterial blood pressure, using the SPSS UNIANOVA procedure (using log-transformed T-cell subset percentages).

## RESULTS

### CD25^+^CD39^+^CD134^+^CD4^+^ T Cells Are Proper iTregs and Share Antigen Specificity With Conventional CD4^+^ T cells

**Figure 1.**
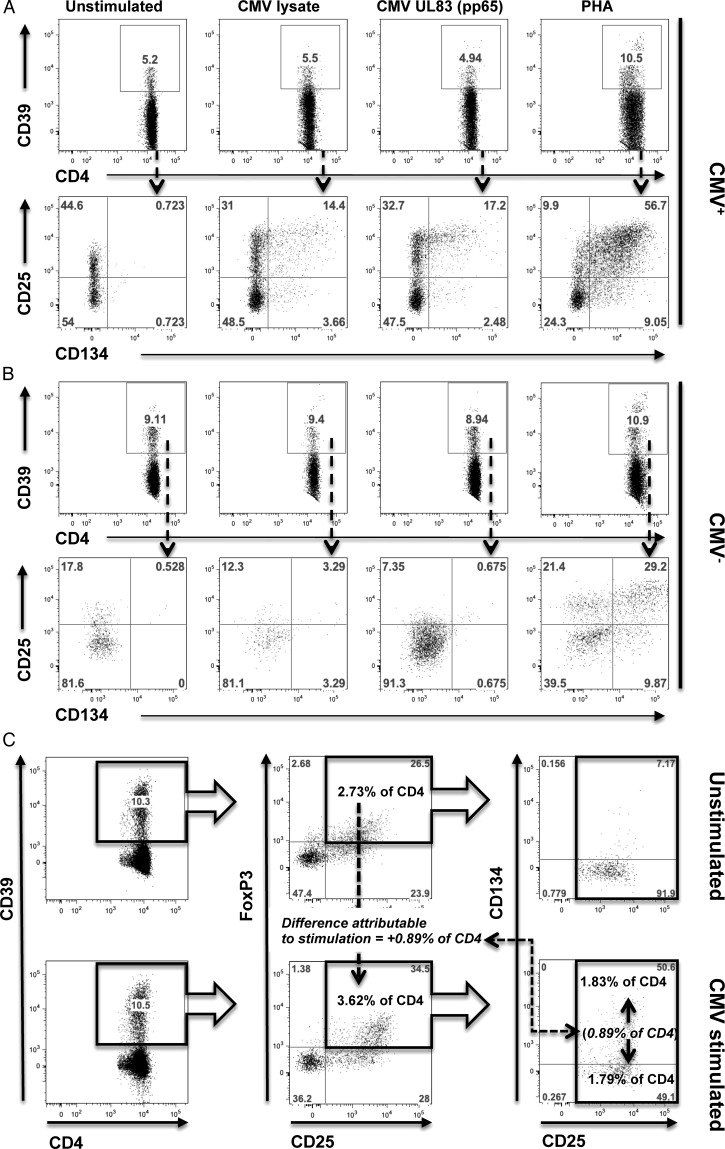
CD25^+^CD39^+^CD134^+^ CD4^+^ T cells result from short term ex vivo activation with specific antigens and coexpress the regulatory T-cell (Treg) marker Foxp3. Panels show experiments in a cytomegalovirus (CMV)–positive donor (*A*) and a CMV-negative donor (*B*). Whole blood was stimulated with medium alone (negative control; unstimulated), CMV lysate, the CMV UL83 peptide pool, and phytohemagglutinin (PHA; positive control). CD39^+^ cells are not significantly increased by CMV lysate or UL83 stimulation but are strongly increased by PHA stimulation. Note that CD134 is upregulated in response to CMV antigens in the CMV-positive participant but not the CMV-negative participant (second row in *A* and *B*). *C*, A representative experiment in an older CMV-positive participant using CMV-lysate stimulation and negative control is shown. Plots show CD4/CD39 staining (left), CD25/Foxp3 staining (middle), and CD134/Foxp3 staining (right). Percentages of relevant subsets as the percentage of CD4^+^ T cells are shown in bold in unstimulated (middle, top), and CMV lysate–stimulated replicates (middle, bottom). The increase of 0.89% of Foxp3^+^ cells in terms of CD4^+^ T cells is the maximum contribution that short-term activation could have made to the Foxp3^+^CD134^+^ subset seen after stimulation (right), accounting for a maximum of 48% of these cells. The results shown are representative of 3 independent experiments.

The expression of CD25 and CD39 (canonical Treg markers) on Tregs before antigen-specific activation is the basis of iTreg detection by the novel method used in this study. Because CD134 is upregulated following CMV-specific activation, the CMV-specific portion of the regulatory subset is revealed as CD25^+^CD39^+^CD134^+^ cells (Figure 1*A* and 1*B* and Supplementary Figure 1). A large proportion of these cells additionally express the hallmark Treg marker Foxp3. Some non-Tregs may express Foxp3^+^ de novo as a result of short-term activation; however, when calculated as the difference in Foxp3^+^ cells between unstimulated and stimulated cells (0.89% of CD4^+^ T cells in the example in Figure 1*C*), these accounted for only about 25% of all Foxp3^+^ cells after stimulation, confirming the majority expressed FoxP3 independently of short-term stimulation.

**Figure 2.**
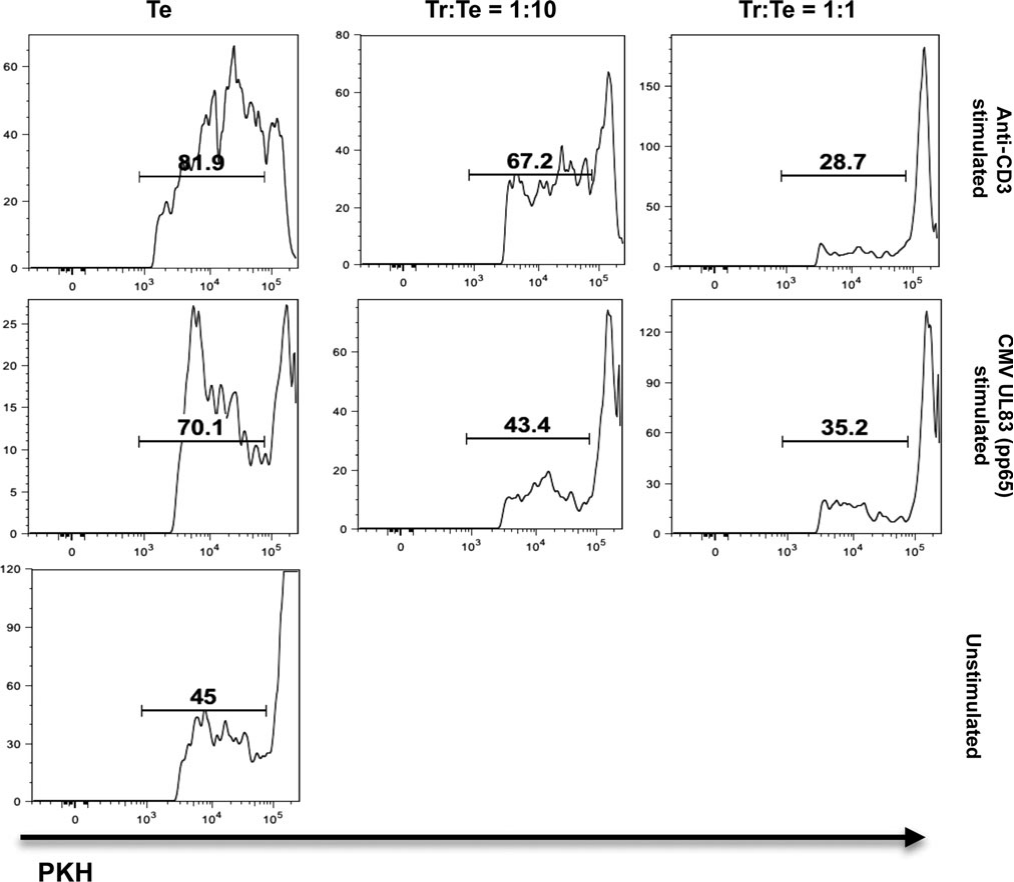
CD39^+^CD25^+^CD134^+^CD4^+^ inducible regulatory T cells (iTregs) are able to suppress autologous CD4^+^ T-cell proliferation. The ability of UL83-stimulated CD39^+^CD25^+^CD134^+^CD4^+^ iTregs to suppress proliferation was tested after sorting them from peripheral blood mononuclear cells by a fluorescence-activated cell sorter and resting them in high-dose interleukin 2 for 2 days. For the suppressor assay iTregs (Tr) were cocultured with PKH67-stained CD4^+^CD25^−^ responder cells (Te) and stimulated with soluble anti-CD3 antibody or UL83 (pp65) peptide pool in the presence of autologous CD3^−^ T cells as antigen-presenting cells. iTregs were added at Tr:Te ratios of 1:1 and 1:10. Responder cell proliferation in the absence of Tregs or in the absence of stimulation were used as positive and negative controls, respectively. Histograms show increasing lipid dye dilution (PKH67) from right to left. Responder cell proliferation was measured as the percentage of cells with decreased PKH67 staining.

**Figure 3.**
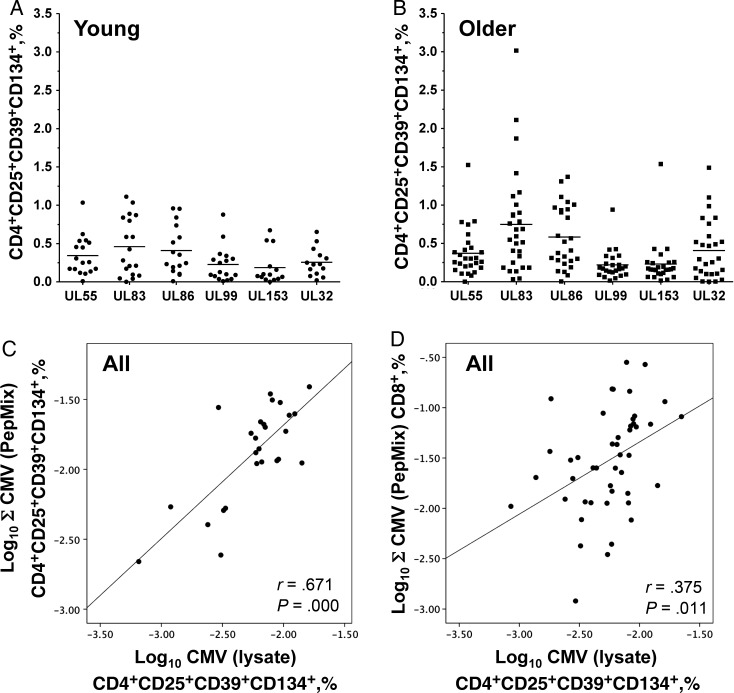
CD39^+^CD25^+^CD134^+^CD4^+^ inducible regulatory T cells (iTregs) are induced by the same antigens that commonly induce effector responses. The induction of iTregs was studied in whole-blood assays of cytomegalovirus (CMV)–positive young donors (*A*) and older donors (*B*) in response to CMV peptide pools including the 6 most dominant CMV CD4^+^ T-cell target antigens (UL55, UL86, UL83, UL32, UL99, and UL153). CMV UL83 (pp65) appeared to elicit the biggest iTreg responses in both young and older participants. *C*, The dot plot shows the relationship between the sum (Σ) of the CMV peptide pool–induced iTreg responses and the CMV lysate–induced iTreg response. *D*, A significant correlation between the CD8^+^ effector T-cell response to CMV (ie, the summated CD8^+^ effector T-cell responses to 19 different CMV proteins) and CMV lysate–induced CD4^+^ iTregs was observed across the whole CMV-positive population sample.

To examine the ability of CD25^+^CD39^+^CD134^+^CD4^+^ T cells to suppress T-cell proliferation ex vivo, we isolated this subset from CMV-stimulated PBMCs, using FACS. In the example (Figure 2), UL83 (pp65)–stimulated PBMCs of a good UL83 responder were sorted into CD25^+^CD39^+^CD134^+^CD4^+^ iTregs and CD25^−^CD4^+^ responder T cells. The isolated iTregs were cocultured with UL83-stimulated or anti-CD3–stimulated autologous responder T cells for 3 days. Both antigen-specific (UL83) and non–antigen-specific (anti-CD3) proliferation was suppressed effectively. The suppressor activity of CMV-induced CD25^+^CD39^+^CD134^+^ iTregs was confirmed in 3 additional donors, providing suppression of the same magnitude. Finally, to explore the protein specificity of CMV-specific iTregs, we studied their responsiveness to the 6 most dominant target antigens of conventional CD4^+^ T cells: UL55, UL86, UL83, UL32, UL99, and UL153 (Figure 3*A* and 3*B*) [28, 30]. All tested peptide pools contributed to the response, and the sum of all responses correlated highly with that induced by CMV lysate (*r* = 0.671; *P* = .000; Figure 3*C*). This suggested that that the pools captured most of the lysate-induced response and that the same antigens that commonly induce effector responses also induce iTreg responses.

To obtain a reliable measure of the total size of the responses of CD4^+^ and CD8^+^ effector T cells to CMV, we summated the responses to 19 different CMV peptide pools (aggregated T-cell response). These 19 pools contained the 6 most important CD4^+^ T-cell antigens listed above and the 15 most important CD8^+^ T-cell antigens (overlapping by UL83 and UL99) as previously described (Table 2) [28]. For CD4^+^ T cells, the CMV lysate–induced response was used in addition. We defined “activated T cells” as T cells displaying at least 1 of the following activation markers: IL-2 production, CD154 upregulation, degranulation, and IFN-γ or TNF-α production [31]. Interestingly, the frequencies of iTregs were correlated with the conventional aggregate CD8^+^ T-cell response (0.375; *P* = .011; Figure 3*D*) but not the CD4^+^ T-cell response to virus lysate (*r* = 0.106; *P* = not significant) or the aggregate CD4^+^ T-cell response (*r* = −0.011; *P* = not significant).

**Table 2.** Effect of Cytomegalovirus-Inducible Regulatory CD4^+^ T Cells (iTregs) on Blood Pressure

Factor	Parameter Estimate (95% CI)	*P*
Mean arterial blood pressure		
Smoker	3.49 (−2.67, 9.64)	.257
Male sex	−38.45 (−91.78 to 14.88)	.151
Age	0.25 (−.14 to .64)	.202
Body mass index	0.19 (−.61 to .99)	.625
Antihypertensive	1.44 (5.19–8.06)	.662
CD4^+^ iTreg count,^a^ per 10-fold increase	20.72 (7.03–34.41)	.004
Sex-associated CD4^+^ iTreg frequency^b^	−18.40 (−41.80 to 4.99)	.119
Diastolic blood pressure		
Smoker	3.09 (−3.54 to 9.72)	.349
Male sex	−62.14 (−119.59 to −4.70)	.035
Age	−0.04 (−.45 to .38)	.858
Body mass index	0.44 (−.42 to 1.30)	.301
Antihypertensive	1.86 (−5.28 to 8.99)	.600
CD4^+^ iTreg count, per 10-fold increase	21.50 (6.75–36.25)	.006
Sex-associated CD4^+^ iTreg frequency^a,b^	−28.07 (−53.27 to −2.87)	.030

Abbreviation: CI, confidence interval.

^a^ T-cell subset percentages were log transformed for linear regression analysis.

^b^ Interaction term.

### CMV-Specific iTreg Frequencies Are Increased at Older Ages, Particularly in Older Women

**Figure 4.**
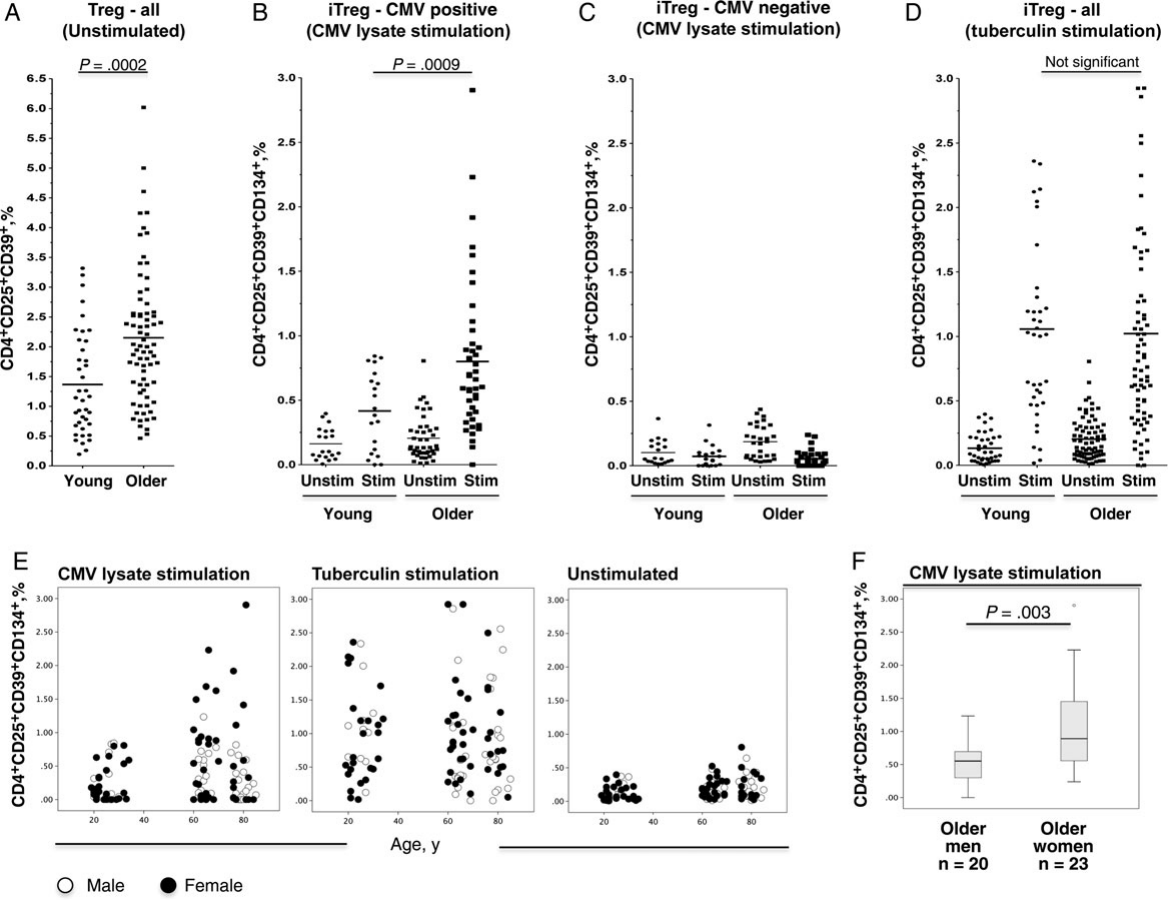
Both CD25^+^CD39^+^CD4^+^ regulatory T cells (Tregs) and cytomegalovirus (CMV)–specific inducible Tregs (iTregs) are significantly expanded in older participants, and their frequencies expose a sex-based difference in older age. Tregs, as well as CMV-specific or tuberculin-specific iTregs, were analyzed by flow cytometry in young and older individuals following ex vivo antigen-specific stimulation; medium alone was used as negative control. *A*, A significant difference in CD4^+^CD25^+^CD39^+^ Treg frequencies between young donors (n = 39) and older donors (n = 74) was observed in the absence of stimulation. *B*, CD25^+^CD39^+^CD134^+^ CMV-specific iTregs were significantly higher in older CMV-positive participants, compared with young CMV-positive participants, following ex vivo antigen-specific stimulation with CMV lysate. *C*, Background levels in CMV-negative participants. *D*, There was no such difference in regard to tuberculin-specific iTregs (including CMV-positive and CMV-negative donors). *E*, When the same analysis was done looking separately at men and women, frequencies were clearly higher in older women than in older men, whereas no significant increase with age-based or sex-based differences was seen with respect to tuberculin-specific iTregs. *F*, The difference in CMV-induced iTregs between CMV-positive older women and men is statistically significant. Abbreviations: Stim, stimulated; unstim, unstimulated.

We initially observed that numbers of CD25^+^CD39^+^CD4^+^ T cells, referred to here simply as Tregs because no specific stimulation was used, were significantly increased at older ages (*P* = .002; Figure 4*A*).The actual extent of CMV-specific iTreg expansion was revealed by CMV lysate stimulation; CMV-responsive but not tuberculin-responsive iTregs were significantly higher in older than young participants (*P* = .009 vs *P* = .478, respectively; Figure 4*B*–*D*). Interestingly, the highest frequencies of CMV-induced iTregs were found in older women. This was not found with tuberculin-induced iTregs (Figure 4*E*), indicating that this highly significant sex-associated difference (Figure 4*F*) was CMV specific. Meanwhile, iTreg induction in the younger group was much smaller, with no significant sex-associated difference (*P* = .230).

Absolute counts of CMV-induced iTregs were not calculated, since the measurements are performed following 44 hours of ex vivo stimulation. To explore a possible association between the relative increase of iTregs in older people and a relative loss of naive CD4^+^ T cells for example, we analyzed the distribution of the CD4^+^ T-cell subsets determined by the expression of classic memory markers, CD27 and CD45RA, in each CMV-positive individual, including naive (CD27^+^/CD45RA^+^), central memory (CD27^+^/CD45RA^−^), effector memory (CD27^−^/CD45RA^−^), and revertant memory (CD27^−^/CD45RA^+^) cells, as previously described [31], and determined their relationship with iTregs in CMV-positive individuals across the population sample (bivariate correlation). The analysis was done for both non–CMV-reactive and CMV-reactive CD4^+^ T cells (ie, those expressing none or at least 1 of 5 analyzed activation markers following CMV-lysate stimulation, respectively). However, none of these correlations proved significant (*r* range, −0.161 to 0.051), suggesting that the relative increase of iTregs is not associated with the relative decline of any specific conventional CD4^+^ T-cell naive/memory subset.

### Frequencies of iTregs and CD8^+^ Effector T Cells Are Associated With Higher Blood Pressure

As a basic measure of vascular status, standardized diastolic blood pressure, systolic blood pressure, and mean arterial blood pressure were determined in all individuals at the time of recruitment. Mean arterial blood pressure was calculated as follows: [diastolic blood pressure] + [0.33 × (systolic blood pressure – diastolic blood pressure)] [32]. Because we wondered whether the immune response to CMV might have an effect on vascular health at older ages, we focused our analysis on CMV-infected older individuals (additional characteristics are available in Supplementary Table 1). Initial exploratory analysis testing simple bivariate correlations observed significant associations between CMV-induced iTregs and mean arterial blood pressure (*r* = 0.330; *P* = .033), as well as diastolic blood pressure (*r* = 0.304; *P* = .05). Slightly stronger associations were found for CD8^+^ effector T cells (*r* = 0.411 [*P* = .018] and *r* = 0.478 [*P* = .005] for mean arterial blood pressure and diastolic blood pressure, respectively). There was no such correlation for CD4^+^ effector T cells, and none of the above T-cell subsets correlated with systolic blood pressure. Interestingly, correlations between CMV-induced iTregs and blood pressure were much stronger in women (*r* = 0.586 [*P* = .003] and *r* = 0.599 [*P* = .003] for mean arterial blood pressure and diastolic blood pressure, respectively) than in men (*r* = 0.104 [*P* = not significant] and *r* = −0.094 [*P* = not significant] for mean arterial blood pressure and diastolic blood pressure, respectively). To allow for a valid and systematic analysis of the size of CMV-specific T-cell subsets on blood pressure, we created a linear regression model including mean arterial blood pressure or diastolic blood pressure as the dependent variable with sex, regular tobacco smoking (any time in the present or past), current hypertensive medication use, and BMI as covariates expected to affect blood pressure. Because CRP is a marker of inflammation, it was unclear whether it was a confounder or mediator, and models were tested with and without adjustment for CRP level. CMV-induced iTregs and CD8^+^ effector cells were significantly correlated both across the whole population sample (Figure 3*D*) and in older people (*r* = 0.403; *P* = .020) and were, therefore, used alternately in the models to avoid collinearity. Linear regression analysis confirmed a significant effect of the size of both T-cell subsets on mean arterial blood pressure (Table 2) and diastolic blood pressure (Table 3). Because adjustment for CRP level had no significant effect on the model, the data shown are unadjusted for CRP level. With regard to mean arterial blood pressure, the interaction between sex and CMV-induced iTregs was not significant; however, this interaction was significant for diastolic blood pressure.

**Table 3.** Effect of Cytomegalovirus-Specific CD8^+^ Effector T cells and Blood Pressure

Factor	Parameter Estimate (95% CI)	*P*
Mean arterial blood pressure		
Smoker	−0.17 (−7.13 to 6.79)	.961
Male sex	2.17 (−4.60 to 8.95)	.513
Age	0.11 (−.33 to .55)	.607
Body mass index	0.43 (−.47 to 1.33)	.335
Antihypertensive	−2.13 (−9.08 to 4.81)	.532
CD8^+^ effector T-cell count,^a^ per 10-fold increase	8.31 (2.20–14.42)	.010
Diastolic blood pressure		
Smoker	0.12 (−7.52 to 7.77)	.974
Male sex	1.02 (−6.42 to 8.46)	.780
Age	−0.17 (−.65 to .32)	.481
Body mass index	0.61 (−.38 to 1.61)	.213
Antihypertensive	−1.94 (−9.57 to 5.70)	.604
CD8^+^ effector T-cell count,^a^ per 10-fold increase	9.51 (2.80–16.22)	.008

Abbreviation: CI, confidence interval.

^a^ T-cell subset percentages were log transformed for linear regression analysis.

## DISCUSSION

This study identified a novel, striking link between CMV-specific cellular immunity and vascular changes in older life. The vast majority of CMV-infected people had CMV-specific CD4^+^ T cells in their peripheral blood that displayed the hallmarks of iTregs and whose frequency was significantly associated with both mean arterial blood pressure and diastolic blood pressure in a linear regression model. The frequencies of CMV-specific CD8^+^ effector T cells were highly correlated with these regulatory-type CD4^+^ T cells and, likewise, significantly associated with mean arterial blood pressure and diastolic blood pressure. These observations point to a direct link between quantitative measurements of CMV-specific immunity and functional vascular parameters. These findings were not explained by confounders such as age, inflammation (ie, CRP level), BMI, smoking history, or use of antihypertensive medication.

The novel CD4^+^ T-cell subset we identified displayed the classic Treg marker Foxp3 to a large extent and suppressed both CMV and anti–CD3-induced proliferation of responder cells, making it a regulatory subset both by phenotype and function. The fact that its antigen specificity was demonstrated by CD134 upregulation convinced us that these cells are a type of CMV-induced iTreg. We further showed that these iTreg responses were dominated by the same antigens as CD4^+^ effector T-cell responses [28], supporting the concept that these are derived from mature CD4^+^ T cells rather than natural Tregs [33].

CMV-specific effector T cells were measured as the aggregate CD8^+^ and CD4^+^ effector T-cell response to 19 different CMV antigens, of which 6 were most relevant for the CD4^+^ effector response and 15 were most relevant for the CD8^+^ effector response, as previously shown [28]. This was an important experimental detail, particularly in terms of CD8^+^ T cells, since the size of the response to single proteins does not generally represent the size of the CMV-specific CD8^+^ T-cell response as a whole [28, 34]. Moreover, CMV lysate is not a good alternative stimulus for CD8^+^ T cells because it does not induce representative CD8^+^ T-cell responses [30].

The association between CMV-specific CD8^+^ T cells and blood pressure was striking from a clinical point of view and in agreement with a published report linking the levels of CMV-specific CD8^+^ T cells with carotid intima media thickness [19]. The observed association between the frequencies of these cells and blood pressure probably reflects the immune response to CMV protein expression in endothelial cells that might result from endothelial infection or antigen delivery by monocytes to sites of vascular injury [8, 11]. Whether the action of CD8^+^ T cells is direct (as in cytotoxicity) or indirect remains to be established, but there is some evidence that T cells can adhere to infected endothelium and that class I MHC is upregulated in CMV-infected endothelial cells [14, 15], both of which would support a direct cell contact-mediated effect. Analogous to what is known from hepatitis B and C, iTregs may be induced (possibly at the expense of conventional CD4^+^ effector T cells) to dampen effector responses directed at CMV-infected endothelium [13]. A recent report that Tregs can attenuate atherosclerosis appears to support this [22], but answering this question definitively will require additional studies.

Interestingly, the marked changes of the immune system in terms of composition and function, previously termed “immune risk phenotype,” include large CD8^+^ T-cell expansions that are believed to be CMV specific to some extent, but this was never conclusively addressed, whereas CMV-induced iTregs were not assessed in these studies [35]. Our findings are, therefore, of major interest, because the main reason why CMV is thought to decrease survival in the older population is its association with increased vascular pathology [3, 4]. Of note, CMV infection has been reported to have additional direct effects on blood pressure, both in humans and in animal models [36, 37]. These seem to be independent of the immune response and may contribute independently to vascular changes in older CMV-infected people.

We currently cannot answer why, in our study, older women had higher numbers of CMV-induced iTregs than older men, but a more successful induction of iTregs in women may contribute to the delay of significant cardiovascular events by driving a more chronic process. As a result of that, we might see increased vascular stiffness (both mean arterial blood pressure and systolic blood pressure correlated with iTregs) but not major damage.

In conclusion, our study provides new and compelling evidence of a quantitative link between CMV-specific cellular immunity and blood pressure or, indirectly, vascular stiffness in older age. We also have identified a novel CMV-specific CD4^+^ T-cell subset with features of Tregs and potential involvement in attenuating effector T-cell responses. Preferential induction of this subset in older women might explain some of the sex-associated differences seen in regard to cardiovascular complications. Together, these findings may indicate that CMV has an important role in driving vascular changes in older life that ultimately affect survival. The level of cellular immunity to CMV might become an important target for intervention in the future, because it is doubtful that the huge CMV-specific T-cell expansions observed in some CMV-infected people [31, 38, 39] are actually required to control infection. Understanding the exact interplay between CMV-specific CD8^+^ T cells, regulatory-type CD4^+^ T cells, and vascular endothelium poses a new and exciting challenge to the field, because cardiovascular pathology remains the leading cause of death worldwide.

## Supplementary Data

Supplementary materials are available at *The Journal of Infectious Diseases* online (http://jid.oxfordjournals.org/). Supplementary materials consist of data provided by the author that are published to benefit the reader. The posted materials are not copyedited. The contents of all supplementary data are the sole responsibility of the authors. Questions or messages regarding errors should be addressed to the author.

Supplementary Data
