# Supplementary material for: A Novel Cytomegalovirus-Induced Regulatory-Type T-Cell Subset Increases in Size During Older Life and Links Virus-Specific Immunity to Vascular Pathology
Source: J Infect Dis. 2013 Nov 7;209(9):1382–92. doi: 10.1093/infdis/jit576 (PMC3982844; doi:10.1093/infdis/jit576)
Supplement: Supplementary Data [file supp_209_9_1382__index.html]

A novel CMV-induced regulatory type T-cell subset increases in older life and links virus-specific immunity to vascular pathology — A Novel Cytomegalovirus-Induced Regulatory-Type T-Cell Subset Increases in Size During Older Life and Links Virus-Specific Immunity to Vascular Pathology — A Novel Cytomegalovirus-Induced Regulatory-Type T-Cell Subset Increases in Size During Older Life and Links Virus-Specific Immunity to Vascular Pathology — A Novel Cytomegalovirus-Induced Regulatory-Type T-Cell Subset Increases in Size During Older Life and Links Virus-Specific Immunity to Vascular Pathology — Supplementary Data 

# A Novel Cytomegalovirus-Induced Regulatory-Type T-Cell Subset Increases in Size During Older Life and Links Virus-Specific Immunity to Vascular Pathology

## Supplementary Data

Supplementary Data

**Files in this Data Supplement:**

- Supplementary Data - Docx file
