# Supplementary material for: A Novel Cytomegalovirus-Induced Regulatory-Type T-Cell Subset Increases in Size During Older Life and Links Virus-Specific Immunity to Vascular Pathology
Source: J Infect Dis. 2013 Nov 7;209(9):1382–92. doi: 10.1093/infdis/jit576 (PMC3982844; doi:10.1093/infdis/jit576)
Supplement: Supplementary Data [file supp_jit576_jit576supp.docx]

**Supplementary Data, Terrazzini et al.**

**Methods**

**Study population**

Exclusion criteria included immunodeficiency (including HIV-infection), organ transplantation, use of immunosuppressive/immunomodulating drugs within the last year, cancer or cancer treatment within the last 5 years, insulin dependent diabetes, moderate/advanced renal failure, liver disease, endocrine disorders (other than corrected thyroid dysfunction), autoimmune disease, dementia/mental incompetence (MMSE <28), alcohol/other drug abuse, acute infection/illness in the last 4 weeks, and raised body temperature. Of the older participants, 38% were taking antihypertensive drugs and/or beta blockers (all of these were classified to be on blood pressure medications), 14% were taking cholesterol-lowering drugs. Of 131 older participants only 3 were smokers at the time of inclusion. A complete past smoking history was provided by 97 of 131 older people including 51 who never smoked and 46 who had smoked in the past (more details are found in supplementary Tables S1 and S2).

**Stimulation of whole blood and PBMC**

Peptide working solution was prepared by dissolving 25 μg per peptide for each the freeze dried pools in 100 μl of DMSO (Sigma-Aldrich) and stored for short-term at 20°C. For each stimulated whole blood tube, 250 μl of complete media containing 1 μg of CMV lysate, 0.5 μg CMV PepMixes, 5 μg of tuberculin or 2.5 μg PHA were added to 0.25 ml of heparin-anti-coagulated whole blood to obtain the final stimulation volume of 0.5 ml. For PBMC stimulation, stimulants and APC-labeled anti-CD107a antibody (BD Biosciences, Oxford, UK) were placed in sterile polystyrene round bottom tubes (Falcon; BD Biosciences) before cell suspension was added. Each tube received 2 μl of dissolved stimulant, 0.5 μl of anti-CD107a antibody, 0.5 μl Monensin (‘Golgistop’, BD Biosciences), and 10^6^ PBMC (200 μl PBMC suspension) plus complete media to a final volume of 250 μl. After 2 hours of incubation at 37°C in 5% CO_2_, humidified atmosphere, 250 μl of Brefeldin A solution (BFA, Sigma-Aldrich) in complete media was added to each tube (final BFA concentration of 10 μg/ml).

Whole blood was incubated in sterile polystyrene round bottom tubes (Falcon, BD Biosciences, Oxford, UK) with caps loosely attached, for 44h at 37^o^C in a humidified 5% CO_2_ atmosphere.

**Antibodies and staining**

Whole blood: After 44 hours of incubation, 100 μl of whole blood was stained with Fluorescein-Isothiocyanate (FITC)-conjugated anti-CD3, Peridinin-chlorophyll-protein complex (PerCp)-conjugated anti-CD4 (both from BioLegend, Cambridge Bioscience, Cambridge, UK), Allophyocyanine(APC)-conjugated anti-CD25, Phycoerythrin (PE)- conjugated anti-CD134, PE-C7-conjugated anti-CD39 (BD Biosciences) for 30min at 4^o^C, followed by treatment with BD FACS lysing solution (according to manufacturer’s instructions) and washed twice with FACS buffer (PBS/0.5% BSA, 0.0.1% sodium azide). For intracellular FoxP3 staining, cells were permeabilized with the Human FoxP3 Buffer Set (BD Biosciences), according to manufacturer’s instructions. Permeabilized cells were stained with Alexa 488-conjugated anti-FoxP3 for 30min at 4^o^C.

PBMC: After 16 hours of incubation, 100 μl 20 mM EDTA was added to each tube and incubation continued for 10min at 37^o^C. Cells were then washed with FACS buffer and stained with v500-conjugated anti-CD3, APC-H7-conjugated anti-CD8, PE-conjugated anti-CD27 (BD Biosciences), Peridinin-chlorophyll-protein complex (PerCp)-conjugated anti-CD4 (BioLegend), ECD-conjugated anti-CD45RA (Beckman Coulter, High Wycombe, UK), live/dead yellow stain (Invitrogen) for 30min at 4^o^C, followed by treatment with BD FACS lysing solution and BD FACS permeabilizing Solution II according to manufacturer’s instructions (BD Biosciences). For intracellular cytokine staining, the following antibodies were used: Pacific Blue (PB)-conjugated anti-CD154, PE-Cy7-conjugated anti-IFN-γ (BioLegend), Alexa 700-conjugated anti-TNF-α, FITC-conjugated anti-IL-2 (BD Biosciences). After 30min incubation at 4^o^C, cells were washed, fixed in 1ml of 0.5% PFA for 5min RT, then washed again and kept at 4^o^C in the dark until acquisition.

**Flow-cytometry analysis**

For the intracellular cytokine staining, separate gates were set for each activation marker on CD4 and CD8 T-cells (IL-2, degranulation, CD154, TNF-α, and IFN-γ). Boolean gates were used to identify functional combinations of markers. The sum of all Boolean gates represents the percentage of cells displaying at least one of the markers. These were termed ‘activated’ effector T cells in this study. Whole blood assays were analyzed with respect to CD25, CD39 and CD134 staining of CD4 T cells where Boolean gates were used for each marker in analogy to the effector cells.

**Supplementary Table S1: Clinical characteristics of older CMV^+^ participants (≥ 60 years) included in the analysis of cellular immunity and blood pressure**

| Variables | No anti-hypertensive medication (incl. beta blockers) | anti-hypertensive medication (incl. beta blockers) | p-Value* |
| --- | --- | --- | --- |
| Female sex, n (%) | 15 (68%) | 8 (38%) | n.s. |
| Male sex, n (%) | 7 (32%) | 13 (62%) |  |
| Age, y | 69.2 ± 7.5 | 70.6 ± 7.7 | n.s. |
| BMI | 26.1 ± 4.0 | 27.9 ± 3.5 | n.s. |
| SP, mm Hg | 143 ± 15 | 144 ± 14 | n.s. |
| DP, mm Hg | 79 ± 11 | 78 ± 9 | n.s. |
| MAP, mm Hg | 100 ± 11 | 100 ± 8 | n.s. |
| oral antihyperglycemic^†^, n (%) | 1 | 1 | n.s. |
| Pos. smoking history, n (%) | 9 | 10 | n.s. |

BMI=body mass index, SP=systolic blood pressure, DP=diastolic blood pressure, MAP = mean arterial pressure, calculated as DP + ((SP-DP)/3)

*Non-parametric test with two-sided significance level p=0.05 or Pearson Chi-Square test, as appropriate; ^†^participants with insulin dependent diabetes were not included in the study

**Supplementary Table S2: Clinical characteristics of older CMV^-^ participants (≥ 60 years) included in the analysis of cellular immunity and blood pressure**

| Variables | No anti-hypertensive medication (incl. beta blockers) | anti-hypertensive medication (incl. beta blockers) | P-Value* |
| --- | --- | --- | --- |
| Female sex, n (%) | 11 (55%) | 5 (45%) | n.s |
| Male sex, n (%) | 7 (47%) | 8 (53%) |  |
| Age, y | 71.0 ± 7.9 | 74.8 ± 8.6 | n.s. |
| BMI | 24.6 ± 4.1 | 27.6 ± 5.9 | n.s. |
| SP, mm Hg | 134 ± 13 | 142 ± 20 | n.s. |
| DP, mm Hg | 78 ± 10 | 79 ± 7 | n.s. |
| MAP, mm Hg | 97 ± 9 | 100 ± 10 | n.s. |
| oral antihyperglycemic^†^, n (%) | 0 | 1 | n.s. |
| Pos. smoking history, n (%) | 6 | 7 | n.s. |

BMI=body mass index, SP=systolic blood pressure, DP=diastolic blood pressure, MAP = mean arterial pressure, calculated as DP + ((SP-DP)/3)

*Non-parametric test with two-sided significance level p=0.05 or Pearson Chi-Square test, as appropriate; ^†^participants with insulin dependent diabetes were not included in the study

**Supplementary Figure S1. Gating strategy for the identification of alternative cell subsets**

The strategy for identifying the different cell subsets determined by the expression of CD25, CD39, and CD134 was based on Boolean combinations of primary positive gates for each of these markers on CD4 T cells (CD3^+^CD4^+^). A representative staining is shown here of unstimulated (top panel) vs CMV-stimulated (bottom panel) whole blood cells.
